# Development and Clinical Application of a Rapid and Sensitive Loop-Mediated Isothermal Amplification Test for SARS-CoV-2 Infection

**DOI:** 10.1128/mSphere.00808-20

**Published:** 2020-08-26

**Authors:** Xuejiao Hu, Qianyun Deng, Junmin Li, Jierong Chen, Zixia Wang, Xiqin Zhang, Zhixin Fang, Haijian Li, Yunhu Zhao, Pan Yu, Wenmin Li, Xiaoming Wang, Shan Li, Lei Zhang, Tieying Hou

**Affiliations:** a Division of Laboratory Medicine, Guangdong Provincial People's Hospital, Guangdong Academy of Medical Sciences, Guangzhou, People’s Republic of China; b MOE International Joint Lab for Synthetic Biology and Medicine, School of Biology and Biological Engineering, South China University of Technology, Guangzhou, People’s Republic of China; c Department of Laboratory Medicine and Central Laboratories, Guangdong Second Provincial General Hospital, Guangzhou, People’s Republic of China; d Genskey Biotechnology Co. Ltd., Beijing, People’s Republic of China; U.S. Centers for Disease Control and Prevention

**Keywords:** RT-LAMP, SARS-CoV-2, COVID-19, asymptomatic carriers, clinical diagnosis

## Abstract

The severe acute respiratory syndrome coronavirus 2 (SARS-CoV-2) outbreak urgently necessitates sensitive and convenient COVID-19 diagnostics for the containment and timely treatment of patients. We aimed to develop and validate a novel reverse transcription–loop-mediated isothermal amplification (RT-LAMP) assay to detect SARS-CoV-2. Patients with suspected COVID-19 and close contacts were recruited from two hospitals between 26 January and 8 April 2020. Respiratory samples were collected and tested using RT-LAMP, and the results were compared with those obtained by reverse transcription-quantitative PCR (RT-qPCR). Samples yielding inconsistent results between these two methods were subjected to next-generation sequencing for confirmation. RT-LAMP was also applied to an asymptomatic COVID-19 carrier and patients with other respiratory viral infections. Samples were collected from a cohort of 129 cases (329 nasopharyngeal swabs) and an independent cohort of 76 patients (152 nasopharyngeal swabs and sputum samples). The RT-LAMP assay was validated to be accurate (overall sensitivity and specificity of 88.89% and 99.00%, respectively) and diagnostically useful (positive and negative likelihood ratios of 88.89 and 0.11, respectively). RT-LAMP showed increased sensitivity (88.89% versus 81.48%) and high consistency (kappa, 0.92) compared to those of RT-qPCR for SARS-CoV-2 screening while requiring only constant-temperature heating and visual inspection. The time required for RT-LAMP was less than 1 h from sample preparation to the result. In addition, RT-LAMP was feasible for use with asymptomatic patients and did not cross-react with other respiratory pathogens. The developed RT-LAMP assay offers rapid, sensitive, and straightforward detection of SARS-CoV-2 infection and may aid the expansion of COVID-19 testing in the public domain and hospitals.

**IMPORTANCE** We developed a visual and rapid reverse transcription–loop-mediated isothermal amplification (RT-LAMP) assay targeting the *S* gene for SARS-CoV-2 infection. The strength of our study was that we validated the RT-LAMP assay using 481 clinical respiratory samples from two prospective cohorts of suspected COVID-19 patients and on the serial samples from an asymptomatic carrier. The developed RT-LAMP approach showed an increased sensitivity (88.89%) and high consistency (kappa, 0.92) compared with those of reverse transcription-quantitative PCR (RT-qPCR) for SARS-CoV-2 screening while requiring only constant-temperature heating and visual inspection, facilitating SARS-CoV-2 screening in well-equipped labs as well as in the field. The time required for RT-LAMP was less than 1 h from sample preparation to the result (more than 2 h for RT-qPCR). This study showed that the RT-LAMP assay was a simple, rapid, and sensitive approach for SARS-CoV-2 infection and can facilitate COVID-19 diagnosis, especially in resource-poor settings.

## Abstract (precis)

We developed a visual and rapid reverse transcription–loop-mediated isothermal amplification (RT-LAMP) assay targeting the *S* gene for SARS-CoV-2 infection. The strength of our study was that we validated the RT-LAMP assay using 481 clinical respiratory samples from two prospective cohorts of suspected COVID-19 patients and on the serial samples from an asymptomatic carrier. The developed RT-LAMP approach showed an increased sensitivity (88.89%) and high consistency (kappa, 0.92) compared with those of reverse transcription-quantitative PCR (RT-qPCR) for SARS-CoV-2 screening while requiring only constant-temperature heating and visual inspection, facilitating SARS-CoV-2 screening in well-equipped labs as well as in the field. The time required for RT-LAMP was less than 1 h from sample preparation to the result (more than 2 h for RT-qPCR). This study showed that the RT-LAMP assay was a simple, rapid, and sensitive approach for SARS-CoV-2 infection and can facilitate COVID-19 diagnosis, especially in resource-poor settings.

## INTRODUCTION

The skyrocketing COVID-19 outbreak has become a public health emergency of international concern. A total of 18,354,342 confirmed cases and 696,147 deaths have been reported in 133 countries since early December of 2019, as of 5 August 2020, according to the WHO COVID-19 report (1). At present, no effective drugs or vaccines have been reported for COVID-19, and prompt diagnosis, close contact tracking, and quarantine management are the hallmarks for the containment of this new pandemic.

Early and accurate diagnosis of severe acute respiratory syndrome coronavirus 2 (SARS-CoV-2) infection is crucial to prevent virus transmission and provide appropriate treatment for patients. Due to its nonspecific symptoms and radiological features overlapping those of the common cold and influenza, the confirmation of SARS-CoV-2 infection depends entirely on viral RNA detection (2, 3). Reverse transcription-quantitative PCR (RT-qPCR) is the standard and most widely used method for SARS-CoV-2 RNA detection in clinical laboratories (4). Despite its outstanding analytical performance, RT-qPCR-based approaches to detect COVID-19 still suffer from many limitations, such as long turnaround times (2 to 4 h), poor availability (it is currently restricted to public health laboratories), the need for expensive instrumentation, and a high proportion of false-negative results or equivocal values (up to 38%) (5, 6) in upper respiratory samples due to insufficient viral materials. These limitations render the RT-qPCR test far from adequate to meet the current challenge of a tremendous undocumented infected population, asymptomatic transmission (7), and convalescence with viral RNA conversion (8), highlighting the pressing need for a more rapid, simple, and sensitive approach to quickly identify infected patients in different settings.

Loop-mediated isothermal amplification (LAMP) is regarded as a promising point-of-care test (POCT) due to its advantages of high sensitivity and specificity, rapid reaction, and low laboratory infrastructure requirements (9). Reverse transcription-LAMP (RT-LAMP) is a type of LAMP method used to detect target RNA with the avian myeloblastosis virus (AMV) reverse transcriptase. This approach allows reverse transcription and DNA amplification to be rapidly accomplished at a constant 60 to 65°C temperature in less than 1 h and in one step, and detailed amplification mechanisms were previously reported (10). RT-LAMP results can be detected by visual turbidity or fluorescence in real time, rendering this method a practical near-patient assay. In recent years, RT-LAMP has been widely used in specialized laboratory testing as well as field surveys to identify various pathogens, including Mycobacterium tuberculosis (11), Zika virus (12), Middle East respiratory syndrome coronavirus (MERS-CoV) (13), and SARS-CoV (14). Shirato et al. (13) reported the development of a useful RT-LAMP assay for the diagnosis of MERS that was developed in this manner, with a detection limit of 3.4 copies per reaction and no cross-reactivity with other respiratory viruses. In addition, Hong et al. (14) developed a real-time quantitative RT-LAMP assay for early and rapid diagnosis of SARS-CoV that demonstrated 100-fold greater sensitivity than conventional RT-qPCR assays.

To accelerate clinical diagnostic testing for COVID-19, we conducted a prospective cohort study to develop and validate a novel RT-LAMP assay capable of detecting SARS-CoV-2 RNA for potential use in centralized facilities and point-of-care settings. Moreover, we compared RT-qPCR and RT-LAMP using clinical samples and demonstrated that RT-LAMP had higher sensitivity and cost effectiveness for SARS-CoV-2 detection. To the best of our knowledge, this study is the first to comprehensively assess a rapid RT-LAMP test for both COVID-19 patients and an asymptomatic carrier, the results of which demonstrated that the test has improved diagnostic value over that of current diagnostics for SARS-CoV-2 infection.

## RESULTS

### Development of an RT-LAMP assay.

As described in Materials and Methods, in this study, we developed a rapid and simple RT-LAMP assay to detect SARS-CoV-2 RNA, where positive reactions resulted in a color change from purple to blue due to a decreased magnesium concentration in the presence of extensive Bst DNA polymerase activity, while negative reactions retained the purple color. Figure S3 in the supplemental material shows the overall procedure of the RT-LAMP assay. RT-LAMP primers for COVID-19 were specific and had a 9.14% to 37.56% nucleotide mismatching with SARS, MERS, and other coronavirus sequences (see Table S2). Furthermore, the cross-reactivity experiment results demonstrated that the RT-LAMP assay did not cross-react with other human-pathogenic coronaviruses and common viral pathogens, supporting the specificity of this assay for COVID-19 (see Fig. S4). Dilution experiments with the synthetic SARS-CoV-2 S gene were performed to determine the limit of detection (LOD) of RT-LAMP relative to that of the RT-qPCR assay for the detection of SARS-CoV-2 (see Fig. S5). The observed LOD values for the RT-LAMP and RT-qPCR assays were approximately 1.5 × 10^−8 ^ng per 25-μl reaction solution (i.e., 4.23 copies/reaction) and 1.5 × 10^−7 ^ng/reaction solution (i.e., 42.3 copies/reaction), respectively. The RT-LAMP assay exhibited a 10-fold higher sensitivity than the RT-qPCR assay currently being used in clinical settings, which is similar to the results of previous LAMP-based assays (15, 16).

### Characteristics of the subjects.

We ultimately collected a prospective cohort of 129 patients from Guangdong Provincial People's Hospital (cohort I: 24 COVID-19 patients [37 nasopharyngeal swab samples] and 105 COVID-19 exclusion cases [292 nasopharyngeal swabs]) and an independent cohort of 76 patients from Guangdong Second Provincial General Hospital (cohort II: 28 COVID-19 patients [56 nasopharyngeal swabs] and 48 non-COVID-19 patients [96 nasopharyngeal swabs]).

The laboratory-confirmed COVID-19 patients had a median age of 46.5 years (interquartile range [IQR], 31 to 60 years), 69.23% (36/52) were male, and most of the patients reported an exposure history and presented primarily with fever, cough/expectoration, and muscle pain/fatigue (Table 1). Most of the COVID-19 patients (94.23%) were identified as nonsevere cases, and only 3 patients were severe cases on admission. Forty of all 52 COVID-19 patients (76.92%) manifested with chest computed tomography (CT) imaging abnormalities, with the most common chest CT patterns being ground-glass opacities (53.85%) and bilateral patchy shadowing (38.46%). The remaining 12 (23.07%) cases showed normal CT images. Twenty-one (40.38%) patients had comorbidities, 15.38% of whom had hypertension and 7.69% had diabetes. Forty (76.92%) patients presented with hematologic abnormalities. The demographic and initial clinical characteristics of the COVID-19 patients in the two cohorts are provided in Table 1.

**TABLE 1:** Clinical characteristics of COVID-19 patients in different cohorts

Clinical features	Value		
	Cohort I (*n* = 24)	Cohort II (*n* = 28)	All patients (*n* = 52)
Sex (no. male/no. female)	16/8	20/8	36/16
Age (yrs) (median [IQR])	52.50 (30.75–61.00)	42.50 (31.75–51.50)	46.50 (31.00–60.00)
Nationality (*n*)			
Chinese	23	27	50
African	1	1	2
Exposure history (*n* [%])	22 (91.67)	25 (89.29)	47 (90.38)
Symptoms (*n* [%])			
Any	23 (95.83)	25 (89.29)	48 (92.31)
Fever	20 (83.33)	15 (53.57)	35 (67.31)
Cough or expectoration	16 (66.67)	6 (21.43)	22 (59.62)
Muscle pain or fatigue	3 (12.50)	9 (32.14)	12 (42.31)
Sore throat	6 (25.00)	14 (50.00)	20 (38.46)
Shortness of breath	6 (25.00)	5 (17.86)	11 (21.15)
Diarrhea	3 (12.50)	5 (17.86)	8 (15.38)
Rhinorrhea	4 (16.67)	3 (10.71)	7 (13.46)
Headache	1 (4.17)	5 (17.86)	6 (11.54)
Nausea or vomiting	1 (4.17)	3 (10.71)	4 (7.69)
Radiologic findings (*n* [%])			
Abnormalities on CT	14 (58.33)	26 (92.86)	40 (76.92)
Ground-glass opacity	10 (41.67)	18 (64.29)	28 (53.85)
Bilateral patchy shadowing	12 (50.00)	8 (28.57)	20 (38.46)
Local patchy shadowing	5 (20.83)	8 (28.57)	13 (25.00)
Interstitial abnormalities	1 (4.17)	12 (42.86)	13 (25.00)
Comorbidities (*n* [%])			
Any	9 (37.50)	12 (42.86)	21 (40.38)
Hypertension	4 (16.67)	4 (14.29)	8 (15.38)
Diabetes	2 (8.33)	2 (7.14)	4 (7.69)
Coronary heart disease	1 (4.17)	2 (7.14)	3 (5.77)
Bronchitis	3 (12.50)		3 (5.77)
Cancer	2 (8.33)		2 (3.85)
Hypohepatia	1 (4.17)	1 (3.57)	2 (3.85)
Chronic obstructive pulmonary disease		1 (3.57)	1 (1.92)
Kidney injury		1 (3.57)	1 (1.92)
Coinfection			
Any	9 (37.50)	3 (10.71)	12 (23.08)
Mycoplasma	9 (37.50)	1 (3.57)	10 (19.23)
Hepatitis B virus infection		1 (3.57)	1 (1.92)
*Mycobacterium tuberculosis*		1 (3.57)	1 (1.92)
Influenza A	1 (4.17)		1 (1.92)
Clinical classification			
Nonsevere	22 (91.67)	27 (96.43)	49 (94.23)
Severe	2 (8.33)	1 (3.57)	3 (3.57)
Hematologic abnormalities (*n* [%])			
Any	20 (83.33)	20 (71.43)	40 (76.92)
C-reactive protein (>5 mg/liter)	14 (58.33)	12 (42.86)	26 (50.00)
Lymphocytes (<1.1 × 10^9^/liter or >3.2 × 10^9^/liter)	12 (50.00)	5 (17.86)	17 (32.69)
Hemoglobin (<130 g/liter)	3 (12.50)	8 (28.57)	11 (21.15)
Neutrophils (<3.5 × 10^9^/liter or >9.5 × 10^9^/liter)	3 (12.50)	11 (39.29)	14 (26.92)
Leukocytes (<3.5 × 10^9^/liter or >9.5 × 10^9^/liter)	4 (16.67)	5 (17.86)	9 (17.31)
Platelets (<125 × 10^9^/liter)	4 (16.67)	3 (10.71)	7 (13.46)
Lactate dehydrogenase (>250 U/liter)	5 (20.83)	2 (7.14)	7 (13.46)
Interleukin-6 (>7 pg/ml)	4 (16.67)	2 (7.14)	6 (11.54)
Samples collected for SARS-CoV-2 testing (*n*)			
Nasopharyngeal swabs	37	28	65
Sputum		28	28

### Diagnostic potential of the RT-LAMP assay for COVID-19 patients and an asymptomatic carrier.

We first evaluated the clinical application of the RT-LAMP assay on 329 nasopharyngeal specimens from cohort I. Of these 329 nasopharyngeal swabs, 35 swabs were confirmed to be SARS-CoV-2 positive according to the combined criteria of positive test results (28 samples) and next-generation sequencing (NGS) confirmation (7 samples) (Table 2, see also Table S3 and Fig. S6). Thirty-one of 35 clinically positive samples were determined to be positive using the RT-LAMP assay, and 3 of 294 clinically negative samples were observed to show a positive reaction, which were confirmed to be false-positive reactions by NGS. The performance of the RT-LAMP assay was as follows: sensitivity, 88.57% (95% confidence interval [CI], 74.05% to 95.46%); specificity, 98.98% (97.04% to 99.65%); positive predictive value, 91.18% (77.04% to 96.95%); negative predictive value, 98.64% (96.57% to 99.47%); positive likelihood ratio, 86.8 (44.8 to 168.2); and negative likelihood ratio, 0.12 (0.07 to 0.19) (Table 2). Compared with that of the RT-qPCR assay, the RT-LAMP assay had significantly better sensitivity (88.57% versus 80.00%) and comparable specificity (98.98% versus 100%) for the diagnosis of SARS-CoV-2 infection (Table 2). The detection results obtained using the RT-LAMP assay showed good concordance with those obtained using the RT-qPCR assay, with a Cohen’s kappa of 0.89 (0.79 to 1.00), 100% positive predictive agreement, and 98.01% negative predictive agreement. These observations are in line with data reported in studies by Baek et al. (17) and others (15, 16).

**TABLE 2:** Diagnostic performance comparison of RT-LAMP and RT-qPCR for SARS-CoV-2 in different cohorts

Subjects	No. of samples		% (95% CI)^*a*^				PLR (95% CI)	NLR (95% CI)	Agreement
	Clinically positive	Clinically negative	Sensitivity	Specificity	PPV	NPV			
Cohort I^*b*^	(*n* = 35)	(*n* = 294)							Kappa, 0.89 (0.79–1.00); positive predictive agreement, 100%; negative predictive agreement, 98.01%
RT-LAMP			88.57 (74.05–95.46)	98.98 (97.04–99.65)	91.18 (77.04–96.95)	98.64 (96.57–99.47)	86.80 (44.8–168.2)	0.12 (0.07–0.19)	
Positive	31	3							
Negative	4	291							
RT-qPCR			80.00 (64.11–89.96)	100.00 (98.71–100.00)	100.00 (87.94–100.00)	97.67 (95.28–98.87)		0.20 (0.15–0.26)	
Positive	28	0							
Negative	7	294							
Cohort II^*c*^	(*n* = 46)	(*n* = 106)							Kappa, 0.93 (0.77–1.00); positive predictive agreement, 100%; negative predictive agreement, 96.49%
RT-LAMP			89.13 (76.96–95.27)	99.06 (94.85–99.83)	97.62 (87.68–99.58)	95.45 (89.8–98.04)	94.48 (13.23–674.7)	0.11 (0.07–0.16)	
Positive	41	1							
Negative	5	105							
RT-qPCR			82.61 (69.28–90.91)	100.00 (96.50–100.00)	100.00 (90.82–100.00)	92.98 (86.76–96.40)		0.17 (0.14–0.22)	
Positive	38	0							
Negative	8	106							

a PPV, positive predictive value; NPV, negative predictive value; PLR, positive likelihood ratio; NLR, negative likelihood ratio.

b In Cohort I, 35 of 37 nasopharyngeal swabs from 24 COVID-19 patients were confirmed to be SARS-CoV-2 positive according to the criteria of RT-qPCR (28 samples) and NGS confirmation (7 samples) (see Table S3 in the supplemental material).

c In Cohort II, 46 of 56 samples (paired nasopharyngeal swabs and sputum samples) from 28 COVID-19 patients were determined to be SARS-CoV-2 positive accordingly (38 were RT-qPCR-positive, and 8 were NGS-positive) (Table S3).

**FIG 1:**
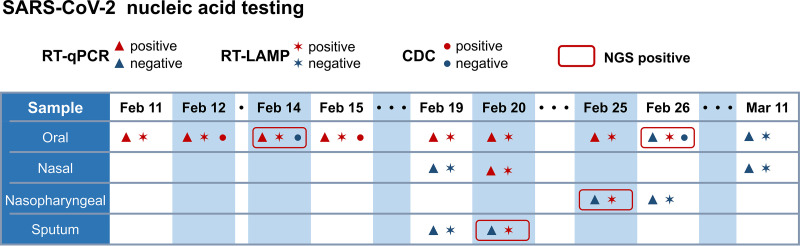
Time line of detection for an asymptomatic COVID-19 carrier.

In addition to exploring the diagnostic potential of RT-LAMP in active COVID-19 patients, we also tested the RT-LAMP assay on an asymptomatic COVID-19 carrier. A 22-year-old female patient presented to our hospital on 13 January 2020 with a 16-year history of congenital heart disease and aggravation of shortness of breath symptoms for 1 month. After admission, she tested positive for SARS-CoV-2 infection by RT-qPCR in our hospital without any COVID-19/viral pneumonia clinical symptoms or CT findings. Her oropharyngeal swabs were also sent to the Guangzhou CDC for repeat RT-qPCR testing and were confirmed to be SARS-CoV-2 positive on 12 February and 15 February. Respiratory samples were collected throughout her illness from 11 February to 11 March and subjected to parallel RT-LAMP and RT-qPCR assays for SARS-CoV-2 detection (Fig. 1). NGS was simultaneously performed for samples yielding inconsistent results between the RT-LAMP and RT-qPCR assays. The number of positive test results obtained by RT-LAMP was 1.37-fold higher than that observed by RT-PCR (11 versus 8), and 4 RT-LAMP-positive but RT-qPCR-negative samples were verified as SARS-COV-2 positive using NGS (Fig. 1 and Table S3). During her hospitalization, the RT-qPCR threshold cycle (*C_T_*) values fluctuated and became negative after 26 February, suggesting a continuous viral shedding pattern and a decreased viral load over time (data not shown, available upon request). This case demonstrated that compared to that of RT-qPCR, RT-LAMP has higher sensitivity in detecting SARS-COV-2, particularly in samples with a low viral load, and also suggested that RT-LAMP can be used for the diagnosis of asymptomatic COVID-19 carriers.

### Validation of the RT-LAMP assay.

We next validated the RT-LAMP assay in an independent cohort (cohort II) of 28 COVID-19 patients and 48 COVID-19 exclusion cases. One nasopharyngeal swab and one sputum specimen were collected from every participant in cohort II. The 152 samples included 46 positive samples (28 swabs and 18 sputum specimens) and 106 negative samples (Tables 2 and S3). Nasopharyngeal swabs from COVID-19 patients showed a higher positive rate than sputum specimens in both the RT-qPCR and RT-LAMP assays (RT-qPCR: swab, 71.43% [20/28]; sputum, 64.29% [18/28]; RT-LAMP: swab, 78.57% [22/28]; sputum, 71.43% [20/28]). The RT-LAMP assay had a sensitivity of 89.13%, whereas that of the RT-qPCR assay was only 82.61%. The specificity of the RT-LAMP assay was roughly equivalent to that of the RT-qPCR assay (99.06% versus 100.00%) (Table 2), and the agreement between the two assays was excellent (kappa, 0.93 [0.77 to 1.00]) (Table 2). These observations corroborate the results obtained from cohort I as well as previous RT-LAMP findings (15–17), suggesting that the use of RT-LAMP may improve the sensitivity of pathogenic diagnosis for COVID-19.

To further assess whether the RT-LAMP assay was specific for COVID-19, 60 swab specimens from 40 patients with influenza (*n* = 14) or other respiratory viral infections (*n* = 26, representing Mycoplasma pneumoniae [MP], human metapneumovirus [HMPV], human parainfluenza virus types II, III, and IV [HPIV-2/3/4], respiratory syncytial virus [RSV], human adenovirus [HAdV], CoV-OC43/229E/NL63, human bocavirus [HBOV], and human rhinovirus [HRV]), and 20 healthy individuals were assessed using the RT-LAMP assay (see Table S4). No positive results were observed, demonstrating that the RT-LAMP-based detection approach can distinguish SARS-CoV-2 with no cross-reactivity for other common respiratory viruses, similar to reports in recent studies (15–17).

**FIG 2:**
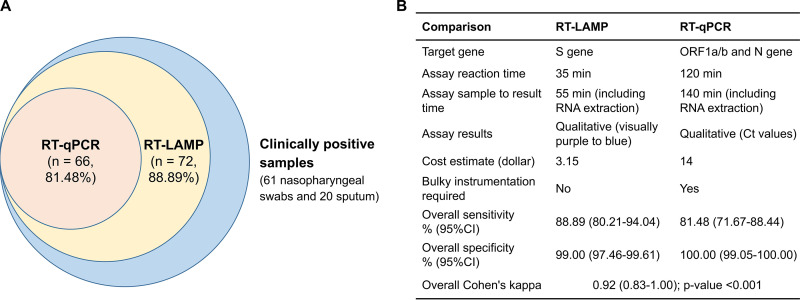
Comparison of the RT-LAMP and RT-qPCR assays for COVID-19 detection in two cohorts. Eighty-one clinically positive samples consisted of 35 nasopharyngeal swabs from cohort I and 46 nasopharyngeal swab and sputum samples from cohort II, which were determined to be positive based on the combined criteria of positive RT-qPCR detection (*n* = 66) or positive NGS confirmation (*n* = 15).

The RT-LAMP assay results reported in this study for SARS-CoV-2 detection in the two cohorts are summarized as follows. The RT-LAMP assay exhibited an overall sensitivity of 88.89% (higher than the 81.48% for RT-qPCR), an overall specificity of 99.00%, high consistency (kappa, 0.92) with the RT-qPCR assay, and a median turnaround time less than 1 h from sample preparation to the result in the detection of 481 clinical specimens from two cohorts (Fig. 2). Additional advantages of RT-LAMP include cost effectiveness, simple operation, and visual determination capability, which facilitate SARS-CoV-2 screening in well-equipped labs as well as in the field (Fig. 2).

## DISCUSSION

Rapid and reliable diagnosis is of particular importance for the containment of COVID-19 outbreaks. In this study, we described a simple and sensitive RT-LAMP approach to rapidly diagnose SARS-CoV-2 infection. The robustness of the present study was demonstrated, as the RT-LAMP assay was useful for the diagnosis of active COVID-19 patients and an asymptomatic carrier and was generally not confounded by other respiratory pathogen infections by using clinical samples from two hospitals.

Existing methods to detect SARS-CoV-2 are primarily based on RT-qPCR, NGS, and IgM and IgG immunological tests. Comparing the results between the RT-LAMP and RT-qPCR assays, RT-LAMP provided better sensitivity (88.89% versus 81.48%) than RT-qPCR for SARS-CoV-2. This added sensitivity is important considering that a significant number of COVID-19 patients have presented with negative qPCR (7) results or the “relapse after negative” phenomenon (8) due to potentially large variability between clinical samples, low-viral-titer samples, and even disrupted binding of RT-qPCR primers due to variation in the viral genome (18). In this study, we used Bst DNA polymerase isolated in-house for the developed RT-LAMP assay, which was demonstrated to have higher polymerization activity than the commercial Bst DNA polymerase (19) and ensured the high sensitivity of this RT-LAMP method. Based on these findings, we propose that the RT-LAMP assay can detect viral RNA not only in samples testing positive by RT-qPCR but also in inconclusive samples.

We observed that the RT-LAMP assay was less sensitive and informative than multiplex PCR-based NGS in our study and other literature (20–24). NGS is a robust tool for obtaining extensive genetic information, allowing LOD values as low as 10 copies/ml for SARS-CoV-2 and serving as a reference test for COVID-19, especially for those challenging samples with a low viral content (2, 22–24). However, several experimental issues, such as erroneous barcode sequencing, the production of primer dimers, and potential cross-contamination between runs, may complicate sequence-based analyses and impact the validity of NGS results (25, 26). Compared to the complex and costly NGS platform, RT-LAMP has the advantages of low-threshold infrastructure, less data processing, and cost effectiveness, enabling this user-friendly assay to be immediately deployed in hospitals and communities. RT-LAMP also showed no cross-reactivity with other viruses that manifest similar respiratory diseases such that the specificity of this assay was higher than that reported for IgM-/IgG-based detection methods (27).

In addition, we described the accuracy of the RT-LAMP assay in detecting SARS-CoV-2 by determining likelihood ratios. Likelihood ratios are not affected by disease prevalence, and values higher than 10 and lower than one strongly support the diagnostic value of a test (28). Based on this metric, the near-patient RT-LAMP assay used in this study is diagnostically useful for COVID-19. Taken together, the RT-LAMP assay established in this study may be a powerful complementary method for monitoring massive numbers of exposed individuals as well as facilitating screening efforts in hospitals and public domains, especially in areas with limited laboratory capacities.

Nasopharyngeal swabs from COVID-19 patients had a higher positive rate than sputum specimens in both the RT-qPCR and RT-LAMP assays. Liu et al. (29) reported that the detection rate of SARS-CoV-2 RNA in nasopharyngeal swabs was lower than that observed in bronchoalveolar lavage fluid and sputum. This inconsistency is most likely due to poor sputum quality and fluctuations in viral RNA levels during different stages of the disease course (30). Despite this inconsistency, nasopharyngeal swabs are noninvasive and easy to acquire, and evidence has shown that SARS-CoV-2 replicates actively in upper respiratory tissue (31). Therefore, we argue that nasopharyngeal swabs are suitable for the detection of SARS-CoV-2 detection at an early stage of infection.

We note that four samples from non-COVID-19 cases tested positive in the RT-LAMP assay but negative by RT-qPCR (Table 2), as reported previously (17). The four false-positive results by RT-LAMP were caused by aerosol contaminants, as we retested these samples in another clean room and obtained the expected negative RT-LAMP result. Contaminant issues are not uncommon for nucleic acid testing, even when the best available reference laboratory tests are used. Precautions to prevent cross-contamination or aerosol contaminants during assays are highly recommended, including the use of a spray solution to eliminate potential RNA fragments and changing gloves frequently. The RNA extraction-free RT-LAMP assay can address this important issue (17). Since this study was completed, the SARS-CoV-2 RT-LAMP test has been optimized further with the use of lyophilized reagents and the direct detection of SARS-CoV-2 without the need for RNA extraction. This one-step single-tube RT-LAMP assay decreases reaction time and minimizes false-positive reactions, making it an ideal POCT for COVID-19 if validated in future studies.

One limitation of our study was the relatively small sample size of positive COVID-19 cases, which resulted in widened confidence intervals for our estimates of diagnostic accuracy. We tested the samples using RT-LAMP in a blind manner, and the designation of the actual status of SARS-CoV-2 infection in clinical samples was based on a set of combined criteria of RT-qPCR results and subsequent NGS confirmation to obviate potential false-negative or false-positive results. We further validated the diagnostic potential of RT-LAMP in another independent cohort with nasopharyngeal swabs and sputum samples. Therefore, despite our small sample size, our study was sufficiently robust for the RT-LAMP assay.

In summary, in this study, we developed a simple and rapid RT-LAMP assay for SARS-CoV-2 detection and demonstrated its high diagnostic sensitivity and specificity among clinical samples. Our findings suggest that RT-LAMP can be an appropriate auxiliary assay for the diagnosis and epidemiologic surveillance of COVID-19 in different hospital and community settings.

## MATERIALS AND METHODS

**FIG 3:**
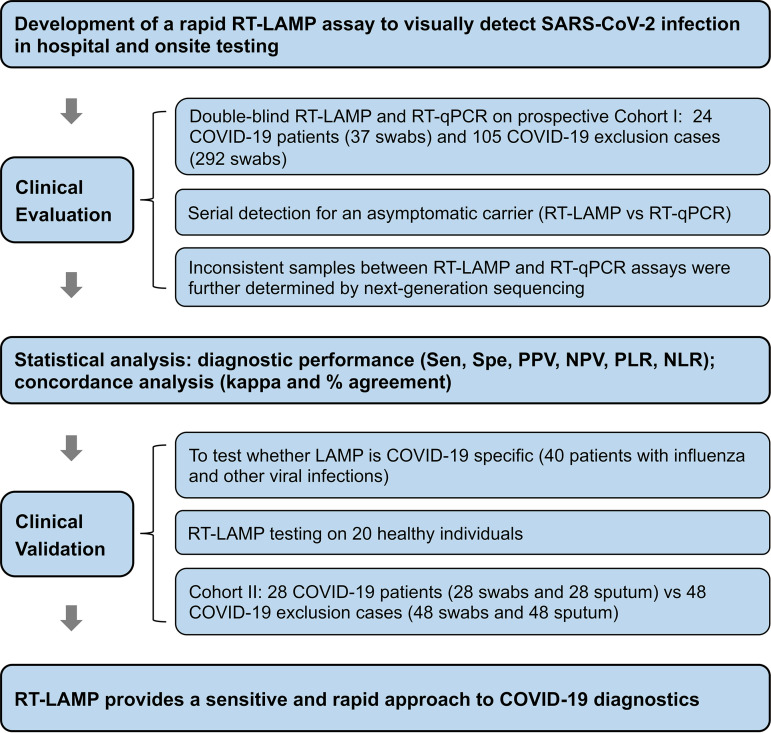
Overview of the study design. Sen, sensitivity; Spe, specificity; PPV, positive predictive value; NPV, negative predictive value; PLR, positive likelihood ratio; NLR, negative likelihood ratio.

This study was designed as a prospective observational cohort study with three sequential phases. In the initial stage, we developed a visual and rapid RT-LAMP assay for SARS-CoV-2 detection and assessed its anti-cross-interface ability, stability, and detection limit. Subsequently, we evaluated the RT-LAMP and standard RT-qPCR assays on 329 nasopharyngeal swabs from a cohort of 129 suspected COVID-19 patients and on serial upper respiratory samples from an asymptomatic carrier, and the inconsistent samples between RT-LAMP and RT-qPCR were further subjected to next-generation sequencing (NGS) for SARS-CoV-2 confirmation. Finally, we analyzed an additional 40 patients with other viral infections, 20 healthy individuals, and an independent cohort of 76 cases suspected of having COVID-19 to further validate the detection capacity of RT-LAMP for SARS-CoV-2. The overall study strategy is shown in Fig. 3.

### Subjects and sample enrollment.

**(i) Cohort I.** Inpatients with clinical-radiological suspicion of COVID-19 presenting to Guangdong Provincial People’s Hospital between 26 January and 8 April 2020, were eligible for inclusion. Close contacts with exposure to confirmed COVID-19 cases were simultaneously enrolled in the present study. Every participant underwent a standard set of SARS-CoV-2 investigations to test COVID-19. The patients’ demographic, clinical, laboratory, and radiological findings were collected from their medical records. Serial nasopharyngeal swabs were collected from patients during hospitalization and close contact screening. The sample sizes for swabs were defined by their availability. At least one nasopharyngeal swab from suspected COVID-19 patients was simultaneously sent to the CDC for double checking as required, where RT-qPCR was routinely utilized for SARS-CoV-2.

COVID-19 was diagnosed based on acute respiratory infection syndromes and/or the presence of chest imaging features consistent with viral pneumonia accompanied by confirmation of positive RT-qPCR test results for SARS-CoV-2 by the CDC, according to the criteria published in the updated COVID-19 diagnostic criteria, 7th edition, China. Suspected COVID-19 patients from Guangdong Provincial People’s Hospital were defined as cohort I in this study and classified into two groups: COVID-19 and non-COVID-19. COVID-19 patients were further classified as nonsevere cases and severe cases; nonsevere cases included patients with mild and moderate pneumonia, and severe cases indicated patients with severe and critically severe acute respiratory distress syndrome (ARDS) or oxygen saturation at rest of <93% who required mechanical ventilation or intensive care unit (ICU) monitoring (2).

**(ii) Cohort II.** We enrolled an independent cohort of suspected COVID-19 patients from Guangdong Second Provincial General Hospital for validation. SARS-CoV-2 testing and the diagnostic procedures for COVID-19 were identical in the two hospitals. A nasopharyngeal swab and 5 ml of morning sputum were collected from suspected COVID-19 patients to validate the diagnostic performance of RT-LAMP for SARS-CoV-2.

In addition, nasopharyngeal swab samples obtained from 20 healthy subjects and 40 patients with other respiratory viral infections were used to test the specificity of RT-LAMP for SARS-CoV-2 detection.

### RNA extraction.

Swabs were preserved in 500 μl of virus preservation solution (TianLong, China), which inactivates viruses and preserves all RNA in the specimen. Sputum samples were preprocessed by standard *N*-acetyl-l-cysteine (NALC)-NaOH digestion. Total RNA was extracted from specimens within 2 h of collection using a magnetic bead-based viral RNA isolation kit with a DA3200 system instrument (Daan Gene, China) according to the manufacturer’s instructions. The extracts were stored at −70°C until use. RNA extracted from each specimen was tested for SARS-CoV-2 in parallel by RT-qPCR and RT-LAMP in a double-blind manner in a biosafety level 2 laboratory. Samples yielding inconsistent results between these two methods were further analyzed by NGS for verification.

### RT-qPCR amplification.

RT-qPCR was performed using an officially approved clinical RT-qPCR kit for the ABI COVID-19 QuantStudio Dx real-time PCR system (Applied Biosystems, USA) according to the manufacturer’s protocol (Daan Gene). Primer and probe sets targeting the *ORF1ab* and *N* genes of SARS-CoV-2 are provided in Table S1 in the supplemental material. For RT-qPCR, each 25-μl reaction mixture comprised 17 μl of reaction buffer, 3 μl of enzyme solution, and 5 μl of template RNA. The cycling program started at 50°C for 15 min for reverse transcription, followed by 95°C for 15 min for PCR initial activation and 45 cycles of 94°C for 15 s and 55°C for 45 s. A cycle threshold value of less than 40 was defined as a positive test. Patients were defined as having laboratory-confirmed COVID-19 when both targets (*ORF1a/b* and *N* genes) yielded positive results, and repeated tests using another approved RT-qPCR kit were necessary for single-target-positive (*ORF1a/b-* or *N*-positive) samples.

10.1128/mSphere.00808-20.7 TABLE S1 RT-qPCR probes and primers for SARS-CoV-2 detection. Download Table S1, DOCX file, 0.1 MB. Copyright © 2020 Hu et al. 2020 Hu et al. This content is distributed under the terms of the Creative Commons Attribution 4.0 International license.

10.1128/mSphere.00808-20.8 TABLE S2 RT-LAMP primers alignment with other coronaviruses. Download Table S2, DOCX file, 0.1 MB. Copyright © 2020 Hu et al. 2020 Hu et al. This content is distributed under the terms of the Creative Commons Attribution 4.0 International license.

10.1128/mSphere.00808-20.9 TABLE S3 NGS results for the samples from patients in the two cohorts and an asymptomatic COVID-19 carrier with inconsistent results between the RT-LAMP and RT-qPCR assays. Download Table S3, DOCX file, 0.1 MB. Copyright © 2020 Hu et al. 2020 Hu et al. This content is distributed under the terms of the Creative Commons Attribution 4.0 International license.

10.1128/mSphere.00808-20.10 TABLE S4 Summary of swab specimens from patients with viral pneumonia and healthy controls using SARS-CoV-2 RT-LAMP and multiplex PCR. Download Table S4, DOCX file, 0.1 MB. Copyright © 2020 Hu et al. 2020 Hu et al. This content is distributed under the terms of the Creative Commons Attribution 4.0 International license.

### RT-LAMP assay.

**(i) RT-LAMP primer design and testing.** The complete genome sequence of SARS-CoV-2 (GenBank accession number MN908947.3) was aligned and compared with the GenBank nucleotide database gene sequences of all species, including other coronaviruses, to identify conserved sequences. A conserved sequence of the *S* gene (nucleotide 22269 to 22494, no. MN908947.3) was selected as the target to design our RT-LAMP primers because it is highly homologous among various COVID-19 sequences and highly divergent from those of other coronaviruses examined. We designed 4 sets of RT-LAMP primers targeting the SARS-CoV-2 *S* gene sequence (no. MN908947.3) using the online PrimerExplorer V5 software (available at https://primerexplorer.jp/e/). One set of RT-LAMP primers with the best parameters was selected, including two outer primers (F3 and B3), two inner primers (forward inner primer [FIP] and backward inner primer [BIP]), and two loop forward (LF) and backward (LB) primers (see Fig. S1), all of which were synthesized by Invitrogen (Shanghai, China). Primer specificity was verified with a BLAST search of the GenBank nucleotide database via comparisons with other coronaviruses and published SARS-CoV-2 sequences, and the percent mismatch results are presented in Table S2.

10.1128/mSphere.00808-20.1 FIG S1 RT-LAMP primers for SARS-CoV-2 detection. RT-LAMP primers included two outer primers (F3 and B3), two inner primers (a forward inner primer [FIP] and a backward inner primer [BIP]), and two loop forward (LF) and backward (LB) primers. FIP consists of the complementary strand of F1c plus F2. BIP consists of B1c plus the complementary strand of B2. Download FIG S1, PDF file, 0.1 MB. Copyright © 2020 Hu et al. 2020 Hu et al. This content is distributed under the terms of the Creative Commons Attribution 4.0 International license.

**(ii) RT-LAMP assay.** For RT-LAMP, each 25-μl reaction mixture comprised 1 μl of 10× primer mix (16 μM [each] FIP and BIP, 2 μM [each] F3 and B3 primers, 4 μM [each] LF and LB primers), 2.5 μl of 10× Isothermal Amplification Buffer Pack (New England Biolabs), 4 μl of 10 mM deoxynucleoside triphosphates (dNTPs), 4 μl of 5 M betaine, 3 μl of MgSO_4_, 2 μl of Bst DNA polymerase (8 U/μl), 1 μl of AMV reverse transcriptase (5 U/μl), 1 μl of 3 mM fluorescent detection reagent (HNB), 5 μl of RNA template, and 2.5 μl of 1‰ diethyl pyrocarbonate (DEPC)-treated H_2_O. The reaction mixtures were incubated in a PCR thermocycler or dry bath at 65°C for 35 min. The optimal incubation condition of 65°C for 35 min was determined based on the banding pattern observed after gel electrophoresis and an absorption spectrum analysis of the RT-LAMP reactions (see Fig. S2). Nontemplate controls (NTCs) were included in each run to ensure the absence of contamination. Positive reactions could be observed by a visual color change from purple to blue, fluorescent light in response to UV excitation, or by the laddering pattern of bands after gel electrophoresis.

10.1128/mSphere.00808-20.2 FIG S2 Absorption spectrum analysis of RT-LAMP amplification to determine the optimal incubation time. (A) Absorption spectrum analysis of RT-LAMP amplification. (B) The timeline of optical density (OD) values at the wavelength at 650 nm. Download FIG S2, PDF file, 0.1 MB. Copyright © 2020 Hu et al. 2020 Hu et al. This content is distributed under the terms of the Creative Commons Attribution 4.0 International license.

10.1128/mSphere.00808-20.3 FIG S3 Schematic overview of the SARS-CoV-2 RT-LAMP testing procedure. A DNA ladder with increasing size was observed on an agarose gel, confirming the expected DNA amplification. Download FIG S3, PDF file, 0.5 MB. Copyright © 2020 Hu et al. 2020 Hu et al. This content is distributed under the terms of the Creative Commons Attribution 4.0 International license.

10.1128/mSphere.00808-20.4 FIG S4 Cross-reactivity evaluation of the RT-LAMP assay. Download FIG S4, PDF file, 0.9 MB. Copyright © 2020 Hu et al. 2020 Hu et al. This content is distributed under the terms of the Creative Commons Attribution 4.0 International license.

10.1128/mSphere.00808-20.5 FIG S5 RT-LAMP and RT-qPCR detection limits for COVID-19 using a series of dilutions of a synthesized *S* gene plasmid. (A) The analytical limit of the RT-LAMP assay by visualization (top) and gel electrophoresis (bottom). M, DNA ladder 2000 marker; N, DEPC-treated water as a negative control. Lanes 1 to 11 refer to 150 to 1.5 × 10^−8^ ng/reaction (i.e., 4.23 × 10^10^ to 4.23 copies/reaction). (B) The analytical limit of the RT-qPCR assay. ΔRn is Rn minus the baseline value, and Rn is the fluorescence of the reporter dye divided by the fluorescence of a passive reference dye. The LOD for the RT-qPCR assay was 1.5 × 10^−7^ ng/reaction (i.e., 42.3 copies/reaction). The negative curve for RT-qPCR is 1.5 × 10^−8^ ng/reaction. The nucleotide 22269 to 22494 fragment of the SARS-CoV-2 Wuhan-Hu-1 complete genome (no. MN908947.3), i.e., the *S* gene (6532), was synthesized by Sangon Biotech Co., Ltd. (Shanghai, China). The SARS-CoV-2 S nucleotide plasmid (6,532 bp) was used for RT-LAMP-and RT-qPCR-based detection. The copy number of nucleotides was calculated using the following formula: nucleotide copies/μl = [nucleotide concentration (g/μl)/(nucleotide length × 327)] × 6.022 × 10^23^. Download FIG S5, PDF file, 1.3 MB. Copyright © 2020 Hu et al. 2020 Hu et al. This content is distributed under the terms of the Creative Commons Attribution 4.0 International license.

10.1128/mSphere.00808-20.6 FIG S6 SARS-CoV-2 genome coverage plot of multiplex PCR-based next-generation sequencing. (A) SARS-CoV-2-positive sample (with full-genome coverage of SARS-CoV-2). (B) SARS-CoV-2-negative sample (only a small region in the SARS-CoV-2 genome could be aligned in the plot). The genome coverage figure shows unique mapping (blue) and multiple mapping (green) read numbers (left *y* axis) on different genome positions of SARS-CoV-2 (*x* axis). Identity (right *y* axis) and the average base similarity of the assembled genome and the reference genome (no. NC_045512.2) are displayed by the black wavy line above. NGS-positive samples were determined based on the following criteria: (i) full-genome coverage of the SARS-CoV-2 genome, and (ii) a median value of SARS-CoV-2 genome depth of ≥10-fold that of the negative control, according to the Genskey Medical Technology’s prior experience in sequencing hundreds of SARS-CoV-2 samples. Download FIG S6, PDF file, 0.1 MB. Copyright © 2020 Hu et al. 2020 Hu et al. This content is distributed under the terms of the Creative Commons Attribution 4.0 International license.

### Cross-reactivity evaluation of the RT-LAMP assay.

Synthesized plasmids of 12 common viral pathogens, including SARS, MERS, influenza A H1N1/H3N2, influenza B, human parainfluenza viruses (HPIV-1/2/3), respiratory syncytial virus (RSV-A/B), Epstein-Barr virus, human cytomegalovirus, human mastadenovirus (HAdV-B/E), enterovirus (EB-U/71), human rhinovirus (HRV-2/14/16), and coxsackievirus (CA16), were used to test potential cross-reactivity in the developed RT-LAMP assay. The RT-LAMP products obtained using these plasmid templates were assayed by 3% agarose gel electrophoresis.

### Detection limit of the RT-LAMP assay.

To determine the lower detection limit of the RT-LAMP assay, samples from a 10-fold gradient dilution series of synthetic SARS-CoV-2 *S* gene cDNA (1.5 × 10^2^ to 1.5 × 10^−9 ^ng/reaction) were used as the template in RT-LAMP reactions, and the minimum concentration of the positive reaction was recorded. This dilution series was assayed in parallel by RT-qPCR using primers targeting the same region of the SARS-CoV-2 genome. The detection limit of the RT-LAMP assay was determined by comparing the lowest concentration of the positive reaction with that obtained by RT-qPCR.

### Multiplex PCR-based next-generation sequencing.

The samples yielding inconsistent results between the RT-LAMP and RT-qPCR assays and those from COVID-19 patients who tested negative by RT-qPCR were further analyzed by multiplex PCR-based enrichment and NGS to detect the SARS-CoV-2 genome. Briefly, total RNA was reverse transcribed to synthesize first-strand cDNA with random hexamers and a Superscript III reverse transcriptase kit (Vazyme, China). Two-step SARS-CoV-2 genome amplification was performed with two pooled mixtures of primer sets (designed by Genskey Medical Technology Co., Ltd.) designed to cover the entire SARS-CoV-2 genome. cDNA was mixed with the components of the first PCR according to the manufacturer’s instructions. The 2nd PCR was performed using the index primers, and the constructed libraries were sequenced on an Illumina NovaSeq paired-end (PE) 150 platform. Data analysis was primarily performed based on an in-house pipeline produced by Genskey Medical Technology. Raw sequences were quality trimmed and subsequently filtered if shorter than 130 bases using fastp v0.19.5. Sequence reads were first filtered against the human reference genome and then aligned to a reference genome of SARS-CoV-2 (NC_045512.2) using Bowtie v2.2.4. The mapped reads were assembled with SPAdes v3.14.0 with kmers ranging from 19 to 109 to obtain the coronavirus genome sequences.

### Statistical analysis.

The sensitivity, specificity, positive and negative predictive values, likelihood ratios, and their respective 95% confidence intervals for the RT-LAMP and RT-qPCR assays of nasopharyngeal specimens were calculated, and agreement analysis was performed using kappa concordance coefficients (a value ≥0.75 was deemed good) and percentage agreement (≥0.9 was considered good) (32). Statistical analyses were performed in the R programming environment.

### Ethics statement.

Written informed consent was obtained from all participants before the study, and the study was approved by the ethics committee of each participating institution. The analysis was conducted on samples collected during standard COVID-19 tests, with no extra burden on patients.
