# Supplementary material for: Development and Clinical Application of a Rapid and Sensitive Loop-Mediated Isothermal Amplification Test for SARS-CoV-2 Infection
Source: mSphere. 2020 Aug 26;5(4):e00808-20. doi: 10.1128/mSphere.00808-20 (PMC7449630; doi:10.1128/mSphere.00808-20)
Supplement: TABLE S1 [file mSphere.00808-20-st001.docx]

**Table S1. RT-qPCR probes and primers used for SARS-CoV-2 detection**

| **Gene** | **Primer** | **Sequence (5’ to 3’)** |
| --- | --- | --- |
| *N* | Probe | FAM-TTGCCCCCAGCGCTTCA-BHQ1 |
|  | Forward | TTGGGGACCAGGAACTAAT |
|  | Reverse | GAAGGTGTGACTTCCATGC |
| *ORF1a/b* | Probe | HEX-TCCCACCCAAGAATAGCATAGATGC-BHQ1 |
|  | Forward | TTTAGATATATGAATTCACAGGGA |
|  | Reverse | ACCAACACCCAACAATTTAAT |
| *RNP* | Probe | Cy5-TCCACAAGTCCGCGCAGAG-BHQ2 |
|  | Forward | AGATTTGGACCTGCGAG |
|  | Reverse | ACTGAATAGCCAAGGTGAG |
