# Supplementary material for: Development and Clinical Application of a Rapid and Sensitive Loop-Mediated Isothermal Amplification Test for SARS-CoV-2 Infection
Source: mSphere. 2020 Aug 26;5(4):e00808-20. doi: 10.1128/mSphere.00808-20 (PMC7449630; doi:10.1128/mSphere.00808-20)
Supplement: TABLE S2 [file mSphere.00808-20-st002.docx]

**Table S2. Alignment of RT-LAMP primers alignment with other coronaviruses**

| **Virus** | **GenBank ID** | | **% Mismatch** | |
| --- | --- | --- | --- | --- |
| COVID-19 | | MN908947 | | 0 |
| Bat SARS-like CoV 2015 | | MG772933.1 | | 21.55 |
| Bat SARS-like CoV 2017 | | MG772934.1 | | 27.87 |
| Bat SARS-CoV RM1/2004 | | KY417144.1 | | 20.2 |
| SARS-CoV ZS-C | | AY395003.1 | | 19.39 |
| Civet-SARS-CoV SZ16/2003 | | AY304488.1 | | 13.49 |
| SARS-CoV | | NC_004718.3 | | 19.38 |
| SARS-CoV MA15 | | FJ882957.1 | | 14.37 |
| Middle East Respiratory CoV | | NC_019843.3 | | 12.42 |
| Betacoronavirus England 1 | | NC_038294.1 | | 9.14 |
| Murine hepatitis virus | | NC_001846.1 | | 28.67 |
| Human Coronavirus 229E | | NC_002645.1 | | 23.51 |
| Human Coronavirus NL63 | | NC_005831.2 | | 19.96 |
| Human Coronavirus HKU1 | | NC_006577.2 | | 26.40 |
| Human Coronavirus OC43 | | NC_006213.1 | | 37.56 |
