# Supplementary material for: Development and Clinical Application of a Rapid and Sensitive Loop-Mediated Isothermal Amplification Test for SARS-CoV-2 Infection
Source: mSphere. 2020 Aug 26;5(4):e00808-20. doi: 10.1128/mSphere.00808-20 (PMC7449630; doi:10.1128/mSphere.00808-20)
Supplement: TABLE S3 [file mSphere.00808-20-st003.docx]

**Table S3. NGS results for the samples from patients in the two cohorts and an asymptomatic COVID-19 carrier with inconsistent results between the RT-LAMP and RT-qPCR assays**

| **Sample ID** | **Specimen Type** | **RT-qPCR** | **RT-LAMP** | **NGS** | **SARS-CoV-2 Positive** |
| --- | --- | --- | --- | --- | --- |
| **Cohort I** |  |  |  |  |  |
| Sample 6 | nasopharyngeal swab | - | + | - | No |
| Sample 8 | nasopharyngeal swab | - | + | - | No |
| Sample 19 | nasopharyngeal swab | - | - | + | Yes |
| Sample 25 | nasopharyngeal swab | - | - | - | No |
| Sample 30 | nasopharyngeal swab | - | + | - | No |
| Sample 41 | nasopharyngeal swab | - | - | + | Yes |
| Sample 56 | nasopharyngeal swab | - | - | + | Yes |
| Sample 84 | nasopharyngeal swab | - | + | + | Yes |
| Sample 106 | nasopharyngeal swab | - | - | + | Yes |
| Sample 143 | nasopharyngeal swab | - | - | - | No |
| Sample 156 | nasopharyngeal swab | - | + | + | Yes |
| Sample 187 | nasopharyngeal swab | - | + | + | Yes |
| **Cohort II** |  |  |  |  |  |
| Sample 341 | nasopharyngeal swab | - | - | - | No |
| Sample 343 | nasopharyngeal swab | - | - | - | No |
| Sample 344 | sputum | - | - | - | No |
| Sample 347 | nasopharyngeal swab | - | - | + | Yes |
| Sample 348 | sputum | - | - | - | No |
| Sample 351 | nasopharyngeal swab | - | + | + | Yes |
| Sample 352 | sputum | - | - | - | No |
| Sample 353 | nasopharyngeal swab | - | + | + | Yes |
| Sample 357 | nasopharyngeal swab | - | - | + | Yes |
| Sample 358 | sputum | - | - | - | No |
| Sample 363 | nasopharyngeal swab | - | - | + | Yes |
| Sample 364 | sputum | - | - | - | No |
| Sample 368 | sputum | - | - | - | No |
| Sample 370 | sputum | - | - | - | No |
| Sample 372 | sputum | - | + | + | Yes |
| Sample 373 | nasopharyngeal swab | - | - | + | Yes |
| Sample 374 | sputum | - | - | - | No |
| Sample 376 | sputum | - | - | + | Yes |
| Sample 391 | nasopharyngeal swab | - | + | - | No |
| **Asymptomatic COVID-19 carrier** | | |  |  |  |
| Sample 503 | nasopharyngeal swab | CDC-, our lab+ | + | + | Yes |
| Sample 510 | sputum | - | + | + | Yes |
| Sample 512 | nasopharyngeal swab | - | + | + | Yes |
| Sample 513 | nasopharyngeal swab | CDC & our lab- | + | + | Yes |

Notes: Samples included RT-PCR-negative samples from COVID-19 patients and RT-PCR-negative/RT-LAMP-positive samples from non-COVID-19 patients. -, negative; +, positive.
