# Supplementary material for: Development and Clinical Application of a Rapid and Sensitive Loop-Mediated Isothermal Amplification Test for SARS-CoV-2 Infection
Source: mSphere. 2020 Aug 26;5(4):e00808-20. doi: 10.1128/mSphere.00808-20 (PMC7449630; doi:10.1128/mSphere.00808-20)
Supplement: TABLE S4 [file mSphere.00808-20-st004.docx]

**Table S4. Summary of swab specimens from patients with viral pneumonia (n = 40) and healthy controls (n = 20) using SARS-CoV-2 RT-LAMP and multiplex PCR**

| **Sample ID** | **Group** | **Number** | **SARS-CoV-2  RT-LAMP** | **Multiplex-PCR** |
| --- | --- | --- | --- | --- |
| Samples 2, 5, 10, 15, 19, 26, 30, 31 | Patients with viral pneumonia | 8 | negative | Influenza Virus A (IFA) |
| Samples 1, 7, 18, 21, 33, 37 | Patients with viral pneumonia | 6 | negative | Influenza Virus B (IFB) |
| Samples 4, 9, 11, 39 | Patients with viral pneumonia | 4 | negative | Respiratory Syncytial Virus (RSV) |
| Samples 6, 17, 40 | Patients with viral pneumonia | 3 | negative | Mycoplasma Pneumoniae (MP) |
| Samples 8, 29, 32 | Patients with viral pneumonia | 3 | negative | Human Rhinovirus (HRV) |
| Samples 25, 34, 38 | Patients with viral pneumonia | 3 | negative | Human Adenovirus (HAdV) |
| Samples 22, 36 | Patients with viral pneumonia | 2 | negative | Human Metapneumovirus (HMPV) |
| Samples 23, 35 | Patients with viral pneumonia | 2 | negative | Human Bocavirus (HBOV) |
| Samples 24, 48 | Patients with viral pneumonia | 2 | negative | Coronavirus OC43 (COV-OC43) |
| Samples 12, 13 | Patients with viral pneumonia | 2 | negative | Coronavirus 229E (COV-229E) |
| Samples 16, 20 | Patients with viral pneumonia | 2 | negative | Human Parainfluenza Virus Type III (HPIV-3) |
| Sample 27 | Patients with viral pneumonia | 1 | negative | Human Parainfluenza Virus Type II (HPIV-2) |
| Sample 14 | Patients with viral pneumonia | 1 | negative | Human Parainfluenza Virus Type IV (HPIV-4) |
| Sample 3 | Patients with viral pneumonia | 1 | negative | Coronavirus NL63 (COV-NL63) |
| Samples 41-60 | Healthy control | 20 | negative | negative |

Notes: Forty swab specimens (Sample 1-40) were collected from 40 patients with other respiratory viral infections, which were confirmed to be positive using a commercial multiplex-PCR assay (Biotron Biotechnology Co., Ltd., China). The multiplex-PCR assay could detect eighteen kinds of common respiratory pathogens in one reaction simultaneously, including IFA, IFB, RSV, MP, HRV, HAdV, HMPV, HBOV, COV-OC43/229E/NL63/HKU1, HPIV-1/2/3/4, SARS-CoV-2, and Chlamydophila pneumoniae (CP).
