# Supplementary material for: Development and Clinical Application of a Rapid and Sensitive Loop-Mediated Isothermal Amplification Test for SARS-CoV-2 Infection
Source: mSphere. 2020 Aug 26;5(4):e00808-20. doi: 10.1128/mSphere.00808-20 (PMC7449630; doi:10.1128/mSphere.00808-20)
Supplement: FIG S1 [file mSphere.00808-20-sf001.pdf]

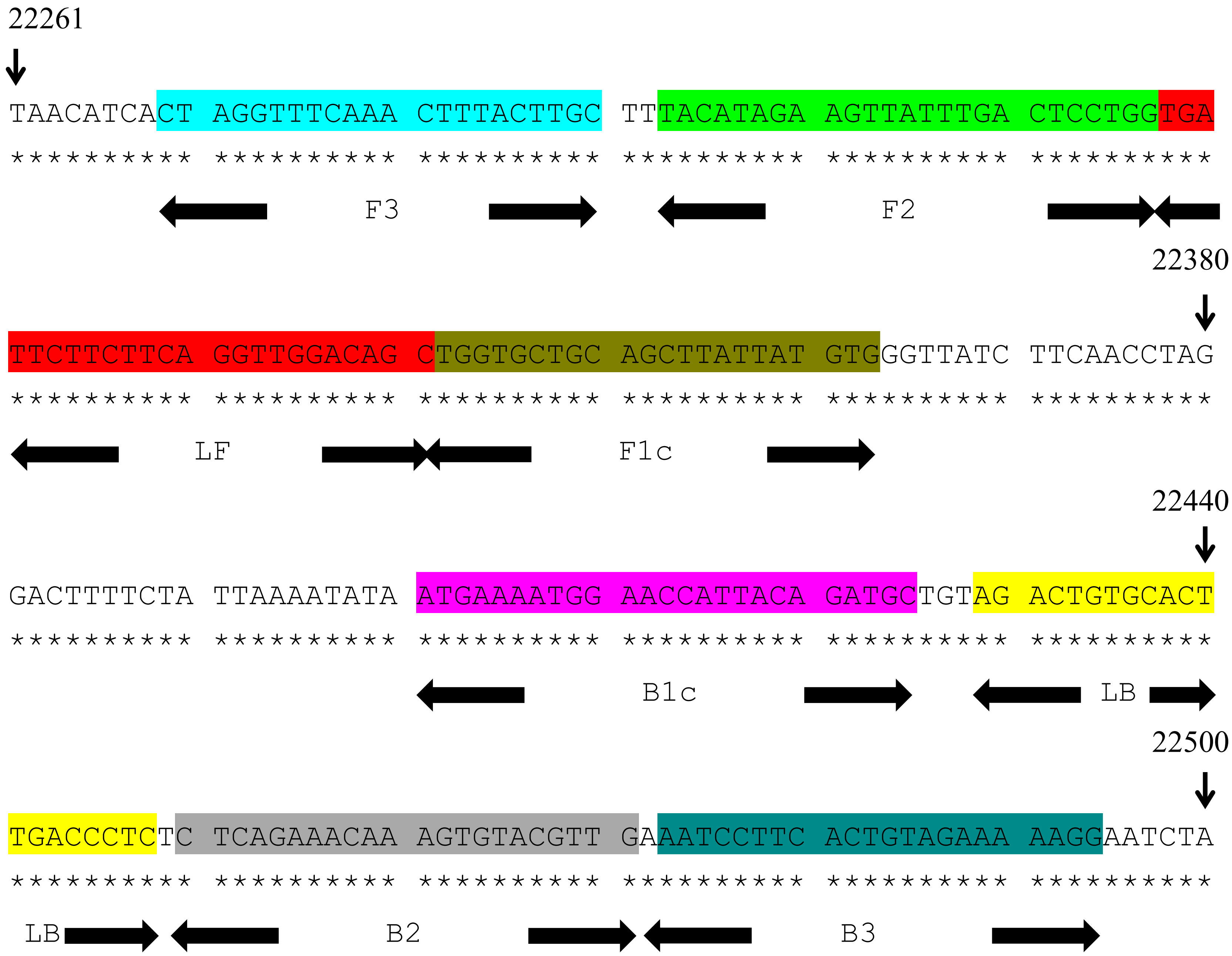
22261
TAACATCACT AGGTTTCAAA CTTTACTTGC TTTACATAGA AGTTATTTGA CTCCTGGTGA
********** ********** ********** ********** ********** **********
F3
F2
22380
TTCTTCTTCA GGTTGGACAG CTGGTGCTGC AGCTTATTAT GTGGGTTATC TTCAACCTAG
********** ********** ********** ********** ********** **********
LF
F1c
22440
GACTTTTCTA TTAAAATATA ATGAAAATGG AACCATTACA GATGCTGTAG ACTGTGCACT
********** ********** ********** ********** ********** **********
B1c
LB
22500
TGACCCTCTC TCAGAAACAA AGTGTACGTT GAAATCCTTC ACTGTAGAAA AAGGAATCTA
********** ********** ********** ********** ********** **********
LB
B2
B3
