# Supplementary figures and images for: Development and Clinical Application of a Rapid and Sensitive Loop-Mediated Isothermal Amplification Test for SARS-CoV-2 Infection
Source: mSphere. 2020 Aug 26;5(4):e00808-20. doi: 10.1128/mSphere.00808-20 (PMC7449630; doi:10.1128/mSphere.00808-20)

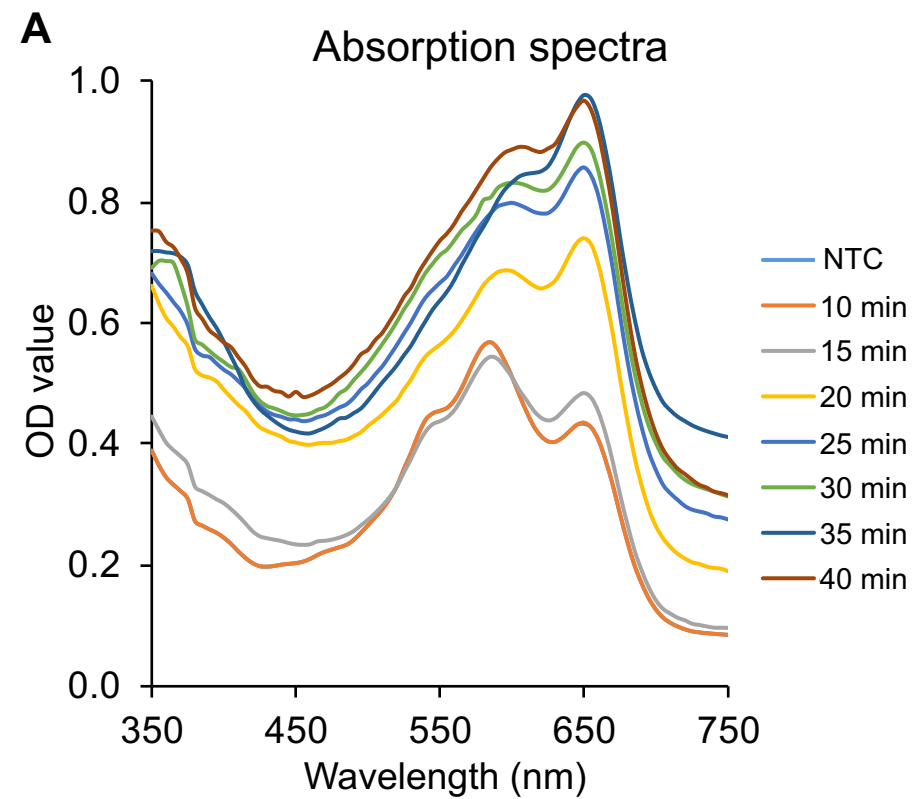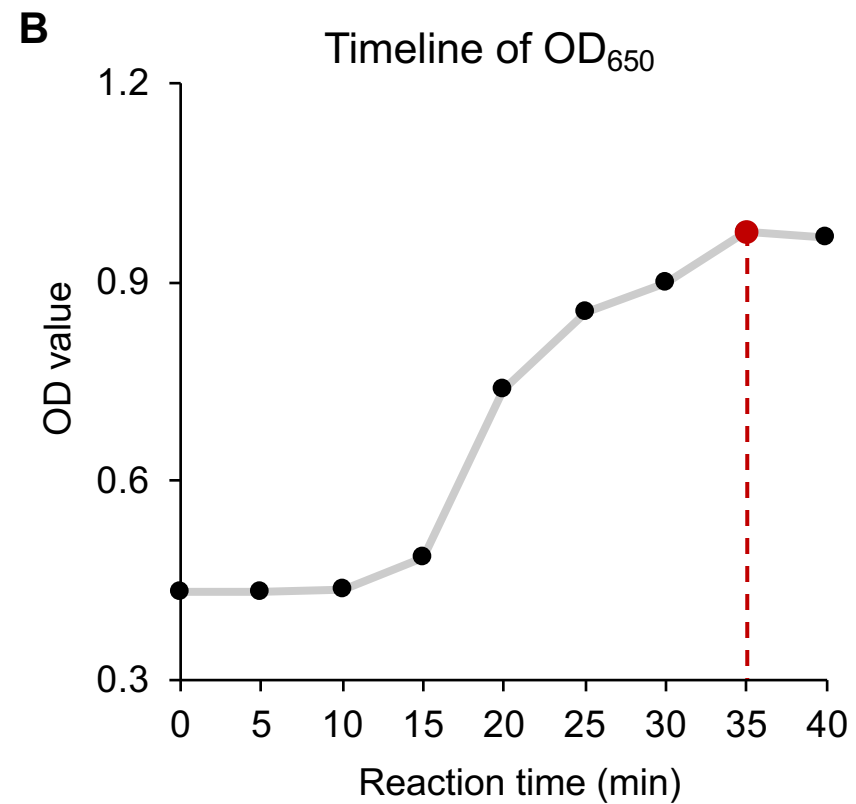

Supplement: FIG S2 [file mSphere.00808-20-sf002.pdf]

**A**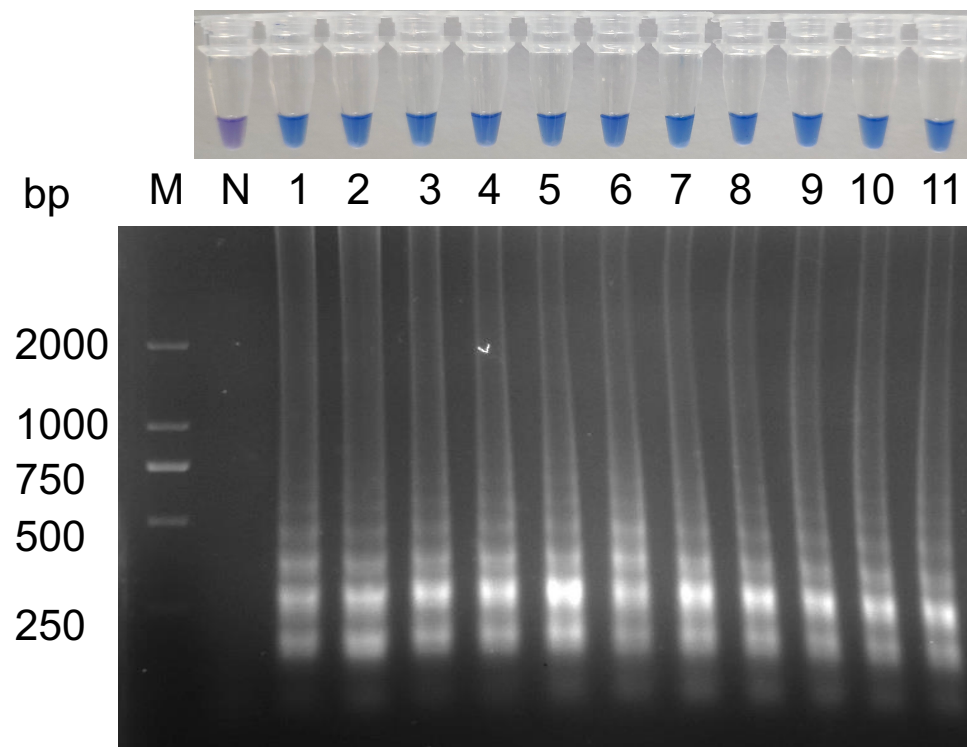**B**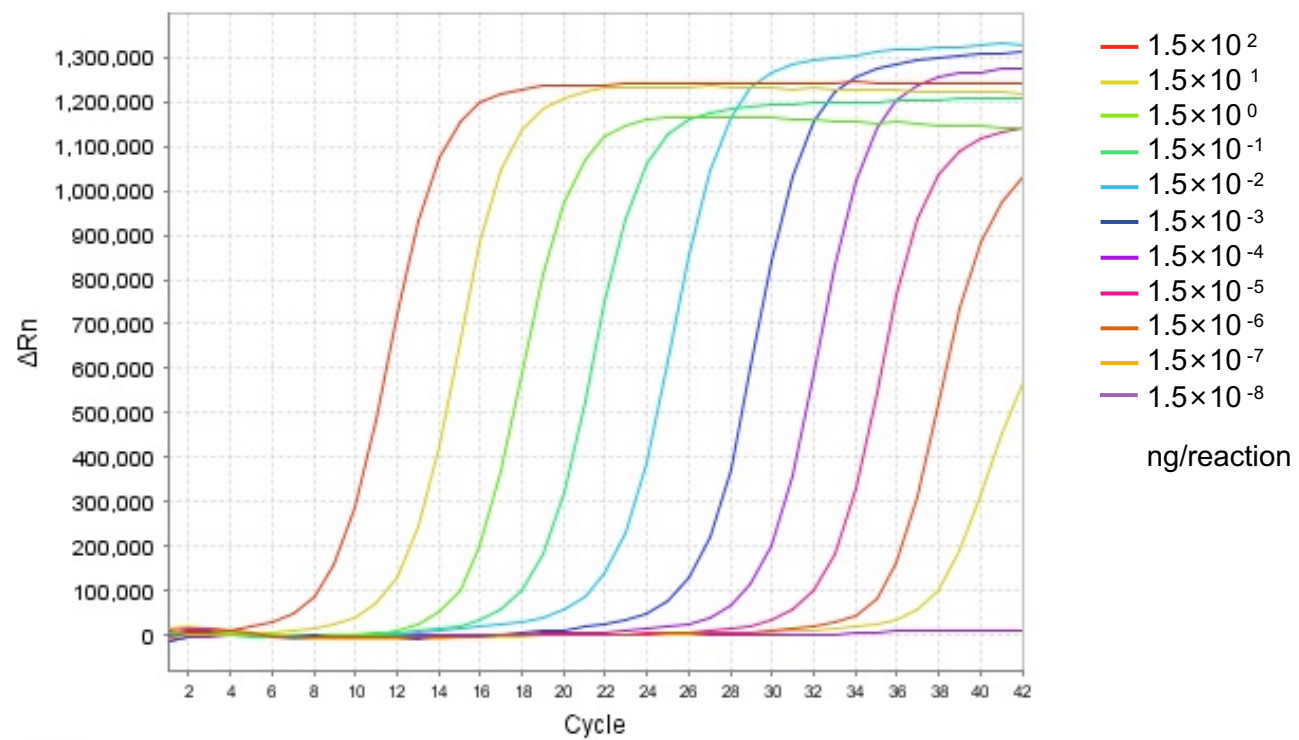

Supplement: FIG S5 [file mSphere.00808-20-sf005.pdf]

**A**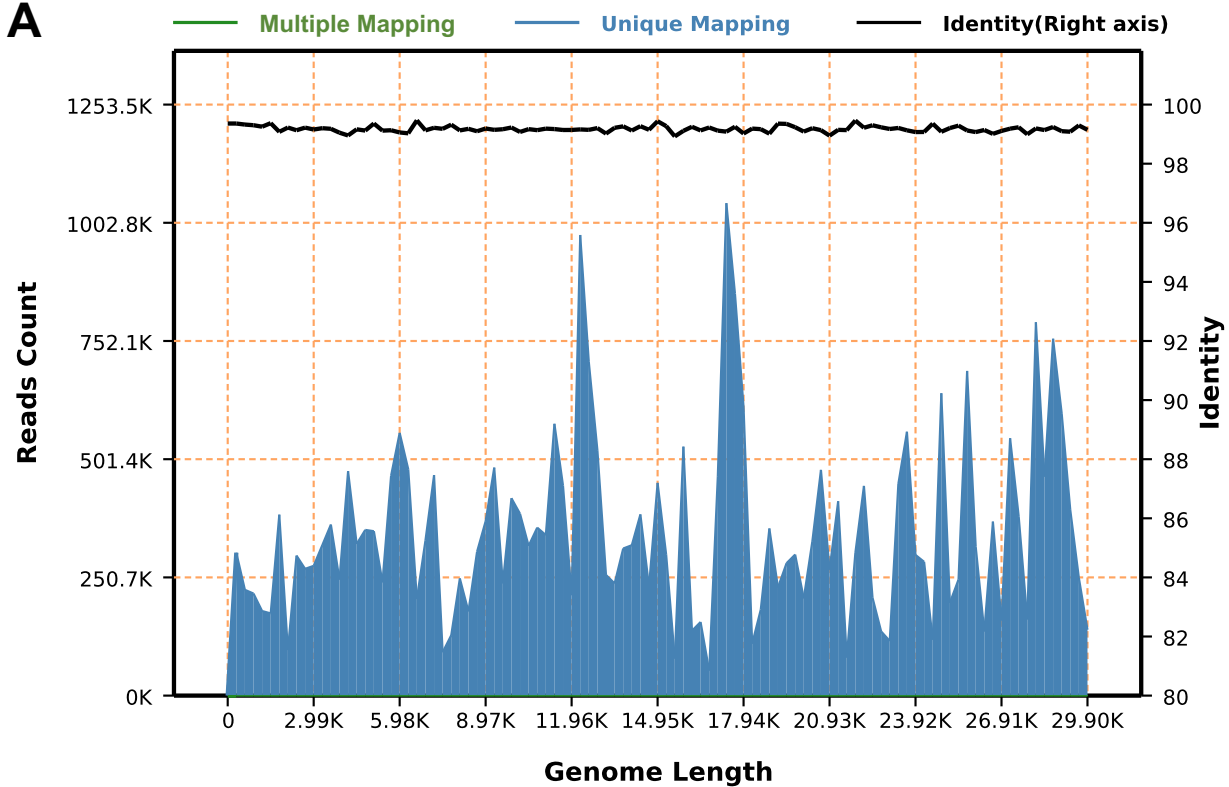**B**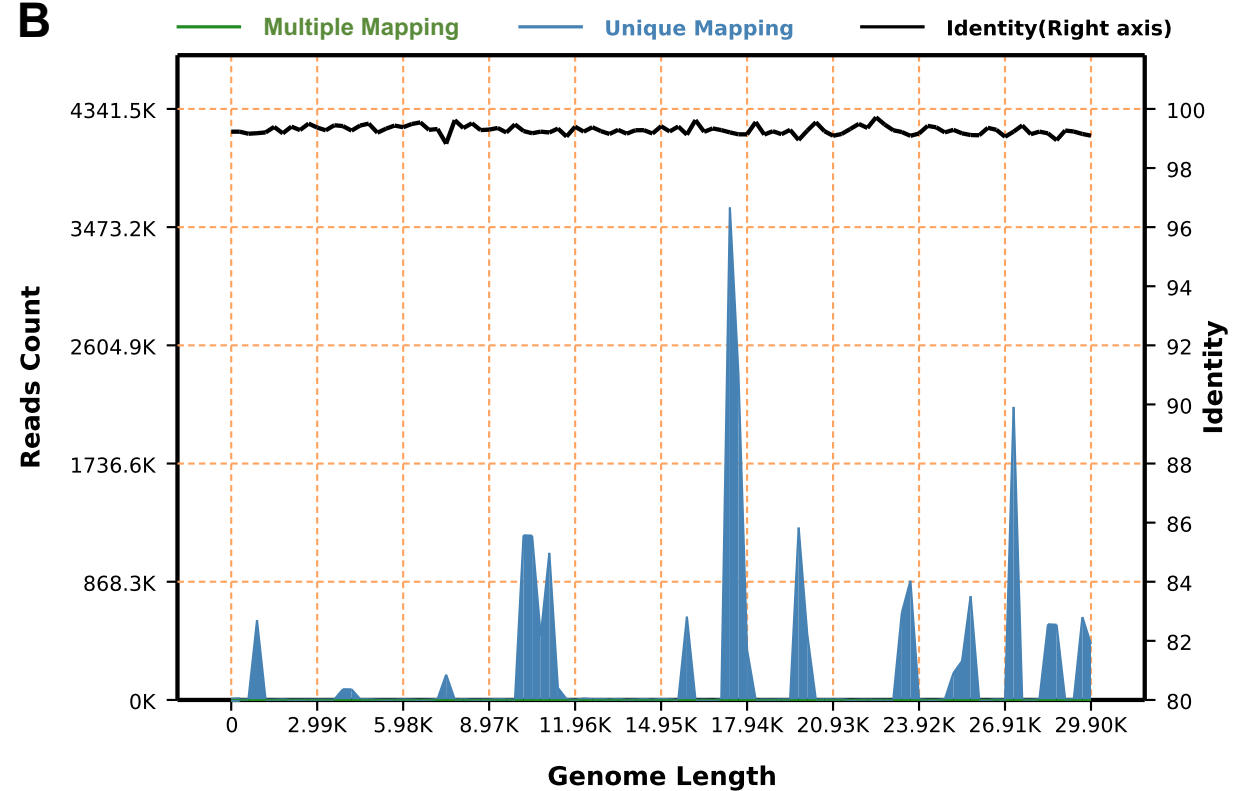

Supplement: FIG S6 [file mSphere.00808-20-sf006.pdf]
