# Supplementary material for: Development and Clinical Application of a Rapid and Sensitive Loop-Mediated Isothermal Amplification Test for SARS-CoV-2 Infection
Source: mSphere. 2020 Aug 26;5(4):e00808-20. doi: 10.1128/mSphere.00808-20 (PMC7449630; doi:10.1128/mSphere.00808-20)
Supplement: FIG S3 [file mSphere.00808-20-sf003.pdf]

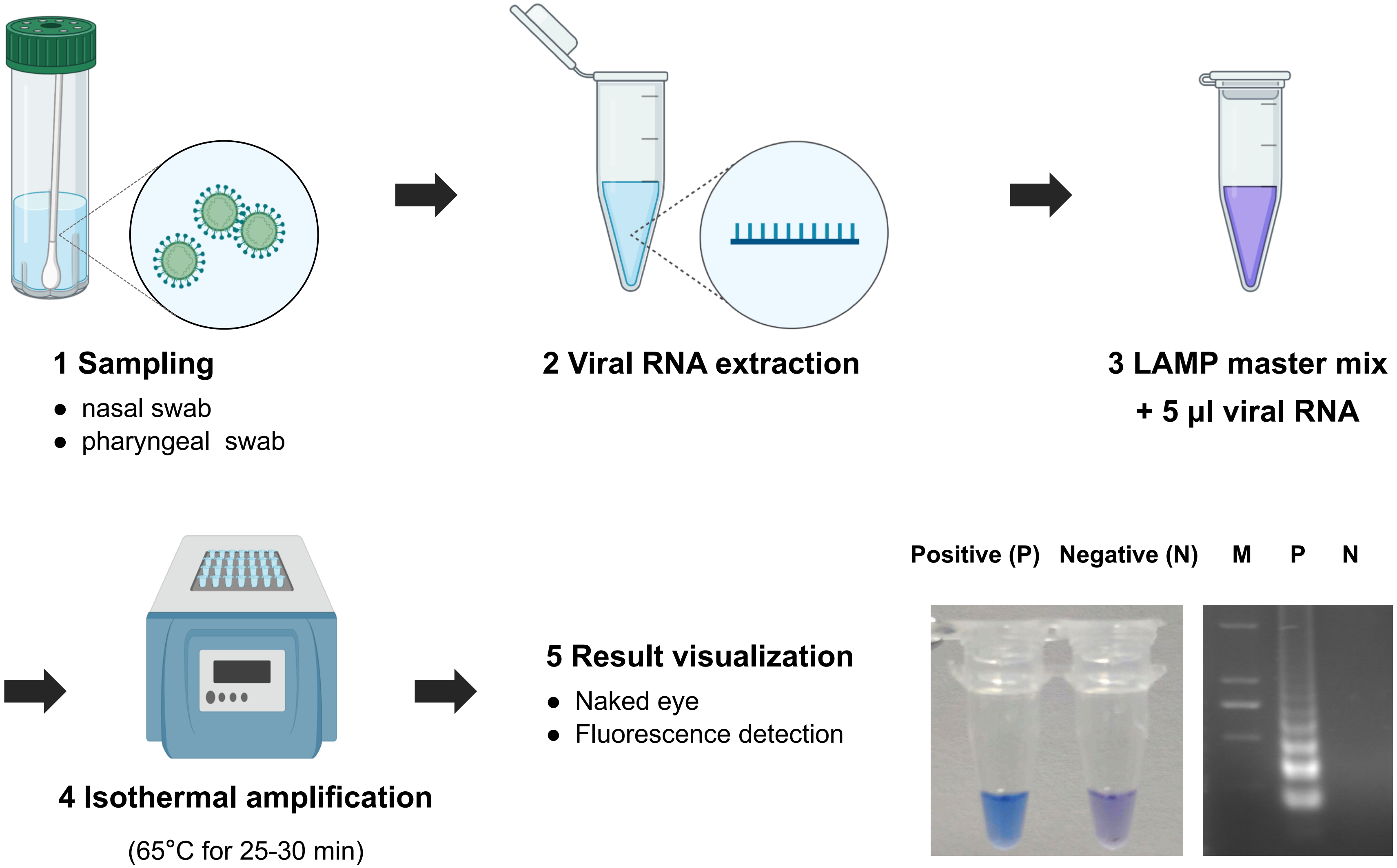
1 Sampling
● nasal swab
● pharyngeal swab
2 Viral RNA extraction
3 LAMP master mix
+ 5 µl viral RNA
4 Isothermal amplification
(65°C for 25-30 min)
5 Result visualization
● Naked eye
● Fluorescence detection
Positive (P)
Negative (N)
M
P
N
