# Supplementary material for: Development and Clinical Application of a Rapid and Sensitive Loop-Mediated Isothermal Amplification Test for SARS-CoV-2 Infection
Source: mSphere. 2020 Aug 26;5(4):e00808-20. doi: 10.1128/mSphere.00808-20 (PMC7449630; doi:10.1128/mSphere.00808-20)
Supplement: FIG S4 [file mSphere.00808-20-sf004.pdf]

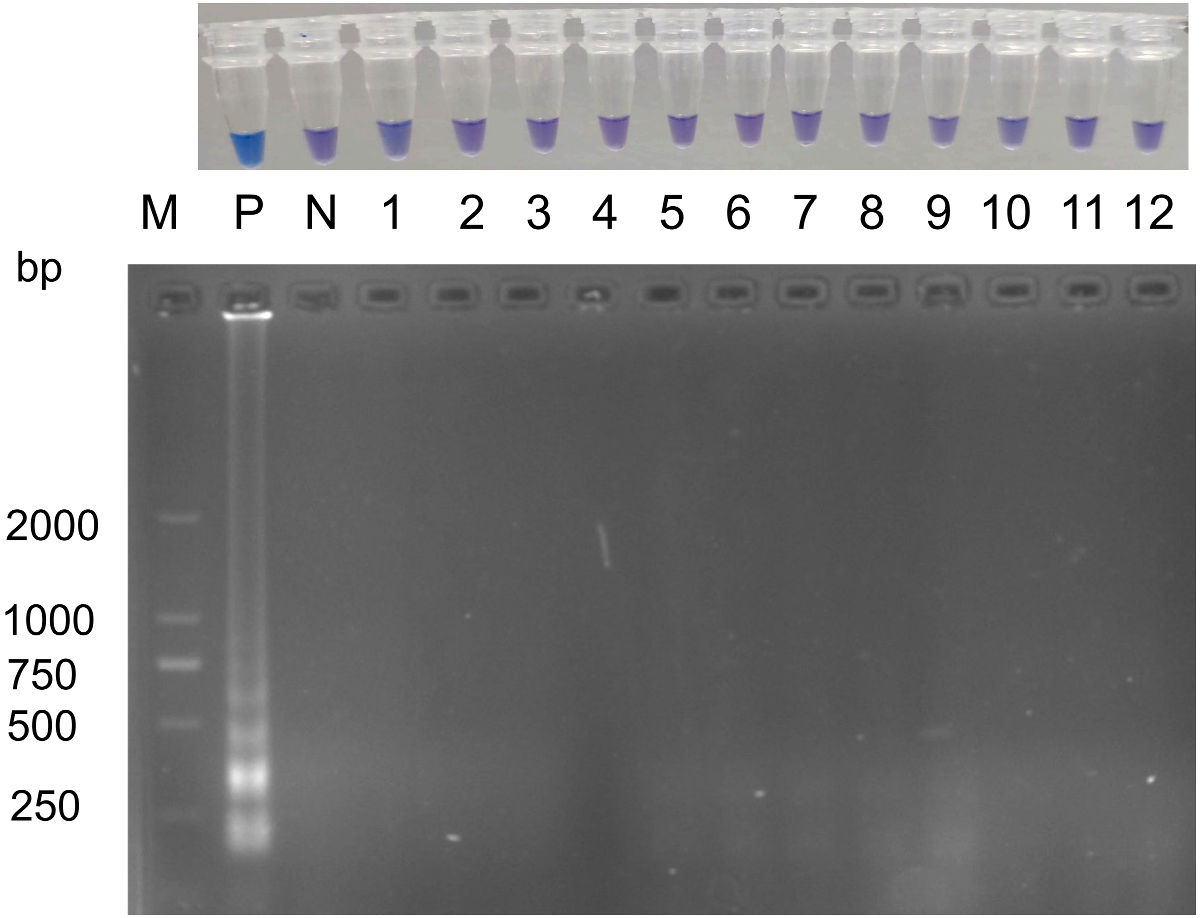

M: Maker; P: positive control; N: negative control
1 - SARS
2 - MERS
3 - Influenza A (H1N1/H3N2)
4 - Influenza B
5 - Human Parainfluenza Viruses (HPIV-1/2/3)
6 - Respiratory Syncytial Virus (RSV-A/B)
7 - Epstein-Barr Virus
8 - Human Cytomegalovirus
9 - Human Mastadenovirus (HAdV-B/E)
10 - Enterovirus (EV-U/71)
11 - Human Rhinovirus (HRV-2/14/16)
12 - Coxsackievirus (CA16)
